# Recovery and prediction of physical function 1 year following hip fracture

**DOI:** 10.1002/pri.1947

**Published:** 2022-03-24

**Authors:** Monica Beckmann, Vigdis Bruun‐Olsen, Are Hugo Pripp, Astrid Bergland, Toby Smith, Kristi Elisabeth Heiberg

**Affiliations:** ^1^ Department of Medical Research Bærum Hospital Vestre Viken Hospital Trust Drammen Norway; ^2^ Institute of Clinical Medicine University of Oslo Oslo Norway; ^3^ Faculty of Health Science OsloMet‐Oslo Metropolitan University Oslo Norway; ^4^ Oslo Centre of Biostatistics and Epidemiology Research Support Services Oslo University Hospital Oslo Norway; ^5^ Faculty of Medicine and Health Sciences University of East Anglia Norwich UK

**Keywords:** elderly, hip fracture, prediction, recovery

## Abstract

**Objectives:**

To investigate the recovery of physical function, health related quality of life (HRQoL), and pain for people following hip fracture for the initial 12 months, and to examine whether postoperative outcome measures of physical function, HRQoL, and pain can predict physical function at 3 and 12 months.

**Design:**

A prospective single‐center observational study, as part of the HIPFRAC trial. Settings: One hospital with two associated municipalities in Norway. Subjects: 207 participants with hip fracture included in the study (140 participants transferred to a short‐term nursing home placement and 67 transferred directly home at discharge from hospital).

**Method:**

Outcome measures were Short Physical Performance Battery (SPPB), Timed Up & Go (TUG), Stair climbing test (SC), Numeric Rating Scale (NRS) for pain at rest and in activity, and EQ‐5D‐5L index and health score. Data were analysed by repeated measures of variance and multivariate regression analyses.

**Results:**

There were statistically significant improvements in physical function (SPPB total score and TUG), NRS‐pain in activity, and HRQoL (EQ‐5D‐5L) from hospital discharge to 3‐month follow‐up for the whole cohort and the two groups (*p* < 0.001). However, the largest improvements occurred within the first 3 months. Further statistically significant improvements occurred between 3 and 12 months (*p* < 0.05). The strongest predictors of physical function at 3 and 12 months post‐fracture were physical function (SPPB) at hospital discharge and pre‐fracture requirement of a walking aid.

**Conclusion:**

The recovery of physical function, HRQoL, and pain in participants after hip fracture indicates gradual improvements during the initial 12‐month follow‐up, with the largest improvements within the first 3 months.

## 1 INTRODUCTION

Patients with a hip fracture often have a medical history which includes physical frailty and low physical capacity (Foss & Kehlet, 2005). Many have reduced mobility and impaired physical function (Kanis et al., 2012; Magaziner et al., 2000; Roth et al., 2010). Physical function as an assessment domain is measured through various approaches and includes mobility (including walking) endurance, muscle strength, and balance (Brovold et al., 2013).

After a hip fracture, patients often experience a considerable deterioration in HRQoL, especially related to self‐care and daily activities (Peeters et al., 2016). Acute post‐operative pain may also have an impact on physical function. Pain is associated with a delay in the initiation of walking during post‐operative rehabilitation (Morrison et al., 2003) and is therefore an important factor for the patients' ability to exercise and their following recovery. The majority never regain pre‐fracture physical function (Bertram et al., 2011). Despite this, patients with hip fracture are discharged from the hospital earlier than before (Deniger et al., 2015; Spehar et al., 2005). In Norway, clinical judgement and assessment of recovery domains whilst patients are in hospital contributes to the decision‐making on whether they can be discharged directly home or to a short‐term placement in a nursing home for a few weeks.

Two key uncertainties existing within the evidence base surrounding hip fracture recovery. First, it is unclear at what time physical function outcomes are maximally recovered. In the study by Magaziner et al. (2000) it was reported that recovery of walking, balance, and strength did not plateau until 9 months post‐injury (Magaziner et al., 2000), while in the study by Dyer et al. (2016) walking capabilities plateaued approximately 6 months post‐fracture (Dyer et al., 2016). Second, there has been limited literature previously reported on potential predictors of physical function after hip fracture. Such predictors have included the Short Physical Performance Battery (SPPB) (Corsonello et al., 2012; Volpato et al., 2011; Xu et al., 2019), handgrip strength (Di Monaco et al., 2015; Xu et al., 2019), and frailty score (Xu et al., 2019). Reduced hand grip strength is associated with falls, disability, reduced HRQoL, prolonged hospital stay and increased mortality (Roberts et al., 2011). SPPB at hospital discharge has been shown to predict physical function up to 12 months following admission in previous cohorts of older people discharged from an acute care hospital settings (Corsonello et al., 2012; Volpato et al., 2011).

Understanding the recovery of physical function, HRQoL, and pain following hip fracture and finding predictors of physical function is of great importance not only to provide insights into the timing and duration of rehabilitation for this patient group, but also for wider acute and rehabilitation care planning following hip fracture (Savino et al., 2013).

The aims of this study are: (1) to investigate the recovery of physical function, HRQoL, and pain for the whole cohort, between those who were transferred to a nursing home placement and those who were discharged directly home for people following hip fracture for the initial 12 months, and (2) to examine whether postoperative outcome measures of physical function, HRQoL, and pain can predict physical function at 3 and 12 months.

## 2 METHODS

### 2.1 Design, setting, participants and ethics

A prospective single‐center observational study was performed in Norway. Data were collected between May 2016 and April 2019 as part of the HIPFRAC trial (Heiberg et al., 2017). This quantitative part of the HIPFRAC trial consists of a randomised controlled trial (RCT) that examined the effect of a health professional‐led functional exercise programme in addition to usual care (usual physiotherapy and usual care) on physical function, HRQoL and pain after 2 weeks in a short‐term nursing home placement and after 3 months (Beckmann et al., 2021). The additional functional exercise programme did not have any effect on physical function, HRQoL and pain, compared to the control group that received usual care alone. This present observational study consists of 3 and 12 months data of patients who needed a short‐term nursing home placement, as well as patients transferred directly home after their hip fracture.

Inclusion criteria were: people aged 65 years or older with low‐energy hip fracture; lived in their own homes pre‐injury in two urban municipalities in Norway; able to walk 10 m with or without a walking aid prior to the fracture; and able to understand both oral and written Norwegian. Participants were excluded if they had a pathological hip fracture or a multi‐trauma injury; had less than 3 months life expectancy; or were unable to take instructions in an assessment situation.

Ethical approval was gained prior to commencement (Committee: South‐East Norway‐Reference: 2015/2147). The study was conducted according to the World Medical Association Declaration of Helsinki (“World Medical Association Declaration of Helsinki: ethical principles for medical research involving human subjects,” 2013).

### 2.2 Data collection

Following informed consent, the participants' baseline data were collected prior to hospital discharge (range: 3–5 days post‐hip fracture) (T1). Data collected included: age, sex, body mass index (BMI), educational level, cohabitant, use of regular medication, comorbidities, type of fracture, surgical treatment of the fracture, the need of walking aid indoors and outdoors, and the need of help from family members and/or community nurses prior to the fracture. Outcome measures of physical function, HRQoL and pain at 3 (T2) and 12 months (T3) post fracture were also included.

Short Physical Performance Battery (SPPB) measured performance‐based physical function (Guralnik et al., 1994). It evaluated balance, walking speed, and lower limb muscle strength. Each sub‐score ranged from 0 to 4, with a total score from 0 to 12 (higher score equate to better performance) (Guralnik et al., 1994). A meaningful clinical change has been reported as 0.5 point (Perera et al., 2006). The SPPB has shown to be valid and reliable for the assessment of older adults (Freire et al., 2012; Guralnik et al., 1994; Perera et al., 2006).

Timed Up & Go (TUG) measured basic functional mobility of frail elderly people (Podsiadlo & Richardson, 1991). The TUG measured the time (in seconds) taken by an individual to stand up from a standard chair, walk a short distance (3 m), turn, walk back to the chair, and sit down. TUG has been reported as a reliable and valid test (Podsiadlo & Richardson, 1991).

Stair‐climbing test (SC) assessed the walking ability to climb stairs. Each participant ascended and descended eight steps as fast as able without running, using alternate legs and support by stair rail if needed. The test was reported in seconds. It has been shown to be reliable and valid among older adults (Ni et al., 2017).

Handgrip strength was measured with a hand dynamometer. Testing was performed with the participant in a sitting position and with the shoulder adducted and neutrally rotated, elbow flexed at 90° with the forearm in neutral position, wrist between 0° and 30° of flexion and between 0° and 15° of ulnar deviation. The best recording of two attempts of maximal voluntary contraction was considered for the analyses. The test has been shown to be reliable and valid among hospitalised older adults (Hillman et al., 2005).

Pain was assessed using an 11‐item Numeric Rating Scale (NRS) for pain. This comprises of a numbered scale from 0 to 10; 0 indicates “no pain”, and 10 indicates the “worst imaginable pain” (Hjermstad et al., 2011). Participants were instructed to choose a single number from the scale that best indicated their level of pain at rest and activity.

Health‐related quality of life was assessed using the EQ‐5D‐5L (“EuroQol‐‐a new facility for the measurement of health‐related quality of life,” 1990). This assesses five dimensions: mobility, self‐care, usual activities, pain/discomfort, and anxiety/depression. Overall health on the day of assessment was also self‐assessed using a 0–100 mm vertical visual analogue scale (EQ‐VAS). Index scores were generated using the Danish scoring algorithm ranging from −0.148 for worst (55,555) to 0.949 for best (11,111) health states (Herdman et al., 2011).

### 2.3 Data analysis

Descriptive data were presented as means and 95% confidence intervals (CI), or percentages. The analyses were conducted per protocol (PP). Missing data were also included in the analyses by using multiple imputations (MI). However, there were no differences in results between PP and MI analyses and accordingly only PP analyses were reported in this paper. Changes in outcome measures were assessed over time, from baseline (T1) to 3 (T2) and 12 months (T3) post‐hip fracture. The dataset was assessed with Kolmogorov–Smirnov test and was mainly normally distributed. Comparison over each time point were analysed using a repeated‐measures analysis of variance (ANOVA).

The differences in outcomes between participants who were discharged to a short‐term nursing home placement compared to directly to home were analysed using independent sample *t* tests at each time point. The associations between the outcome measures and the predictors were analysed using Pearson's correlation analyses. The variables that fulfilled the correlation criteria were included in the multiple regression analysis. The predictors with the smallest contribution to explain the variance of the dependent variable were excluded from the model by a manual backward stepwise procedure. Only the statistically significant predictors (*p* < 0.05) were selected. The regression coefficients were reported with 95% CI. *p‐*values of 0.05 or less were considered statistically significant.

## 3 RESULTS

### 3.1 Participants

**FIGURE 1:**
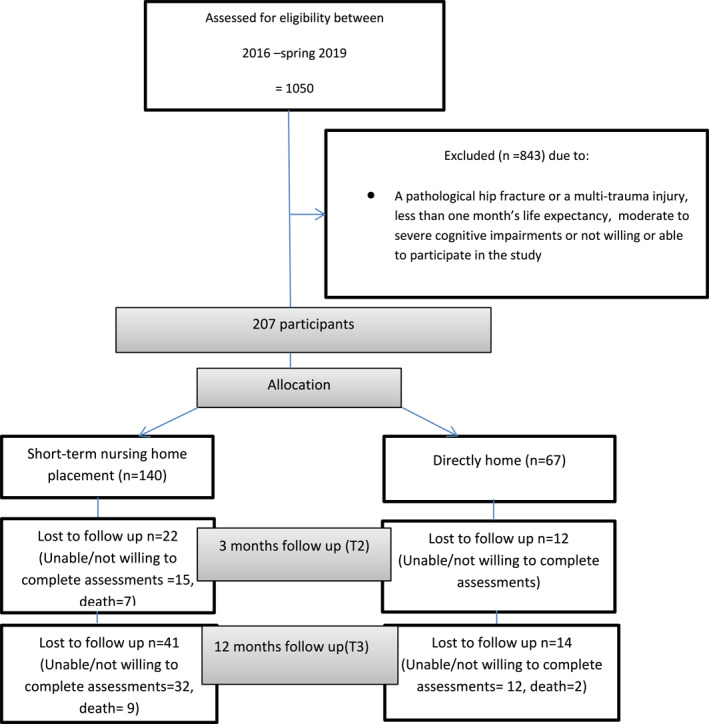
The CONSORT flow chart

In total, 207 participants were recruited; 140 were discharged to a short‐term nursing home placement and 67 directly home. The cohort's baseline characteristics and the differences between groups are presented in Table 1. The follow‐up rates are presented in Figure 1.

**TABLE 1:** Characteristics of the participants with hip fracture at hospital discharge

	Total cohort	Discharged to short‐stay nursing home placement	Transferred directly home	*p* value
*N*	207	140	67	‐
Age, mean years (SD)	82.6 (8.3)	85.0 (7.0)	77.0 (8.2)	<0.001
Sex, women %	76.8	80.6	67.7	0.04
BMI (SD)	23.5 (8.5)	23.5 (10.2)	23.7 (2.7)	0.88
Married %	40.6	33.0	58.0	0.001
University education %	39.7	39.7	40.3	0.40
Osteoporosis %	29.6	34.3	19.4	0.07
Arthrosis %	32.7	31.0	37.1	0.69
Cancer %	20.6	25.5	9.7	0.01
Diabetes %	8.8	9.7	6.5	0.36
Lung disease %	10.2	12.0	4.8	0.03
Heart disease %	25.5	32.6	8.1	<0.001
Neurological disease %	7.4	7.6	6.5	0.79
In need of help from community nurses before the fracture %	25.5	35.4	6.5	0.002
Use of regular medications %	84.0	89.6	69.4	<0.001
In need of a walking aid before the fracture %				
Indoor	33.7	41.7	12.9	<0.001
Outdoor	49.3	61.0	21.0	<0.001
Type of hip fracture (treatment) %				
Fracture colli femoral (two parallel screws)	12.1	14.0	8.1	0.14
Fracture colli femoral (hemiarthroplasty)	54.9	49.0	67.7	0.03
Per trochanteric fracture (dynamic hip screw)	28.6	31.3	22.6	0.04
Sub trochanteric fracture (intramedullary hip screw)	3.9	4.9	1.6	0.30

#### 3.1.1 Recovery of physical function, HRQoL, and pain for the whole cohort

**FIGURE 2:**
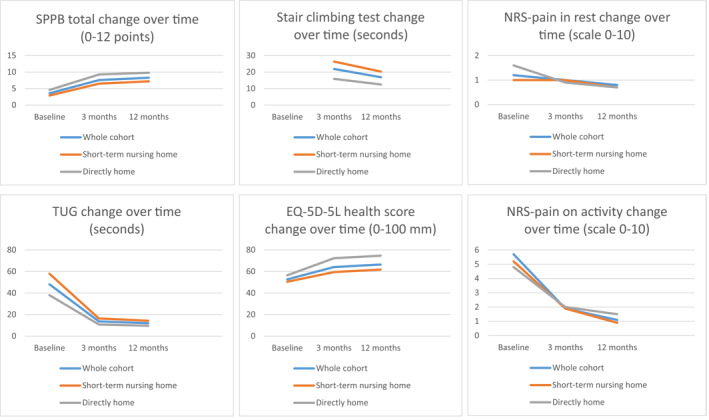
Line graphs of physical health outcomes during the first post‐operative year in participants with hip fracture

There was a statistically significant improvement in SPPB total score (*p* < 0.001) (increased by four points), TUG (*p* < 0.001) (decreased by 34.4 s), EQ‐5D‐5L score (*p* < 0.001) (increased score by 11.4 points) and NRS Pain in activity (*p* < 0.001) (decreased by 3.1 points) between hospital discharge to 3‐month follow‐up. There were no statistically significant improvements in EQ‐5D‐5L Index and NRS Pain at rest during this interval (*p* > 0.05) (Table 2; Figure 2).

**TABLE 2:** Recovery of physical function, quality of life, and pain from baseline (T1) to 3 (T2) and 12 months (T3) in participants after hip fracture (*n* = 207)

Variables	Mean (95% CI)			*p*‐value for:		
	Baseline (T1)	3 months after hip fracture (T2)	12 months after hip fracture (T3)	Overall time effect	Difference from T1 to T2	Difference from T2 to T3
SPPB total, points 0–12	3.6 (3.2, 3.9)	7.6 (7.1, 8.2)	8.3 (7.7, 8.8)	<0.001	<0.001	<0.001
SPPB balance, 0–4	2.2 (2.0, 2.5)	3.0 (2.8, 3.2)	3.0 (2.8, 3.2)	<0.001	<0.001	1.00
SPPB walking speed, 0–4	1.2 (1.1, 1.3)	3.0 (2.8, 3.2)	3.2 (3.0, 3.3)	<0.001	<0.001	0.06
SPPB strength, 0–4	0.1 (0.04, 0.2)	1.5 (1.2, 1.8)	2.0 (1.7, 1.3)	<0.001	<0.001	<0.001
TUG, seconds	48.1 (43.3, 53.3)	13.7 (12.2, 15.2)	12.0 (10.7, 13.3)	<0.001	<0.001	<0.001
Stair climbing test, seconds	‐	21.9 (18.8, 24.9)	16.9 (14.9, 19.0)	<0.001	‐	<0.001
EQ index	0.5 (0.4, 0.5)	0.6 (0.5, 0.6)	0.8 (0.7, 0.8)	<0.001	0.16	<0.001
EQ health score	52.7 (49.4, 55.9)	64.1 (61.2, 67.0)	66.5 (63.0, 69.8)	<0.001	<0.001	0.37
NRS pain at rest	1.2 (0.9, 1.5)	1.0 (0.8, 1.3)	0.8 (0.5, 1.0)	0.005	0.69	0.10
NRS pain in activity	5.0 (4.7, 5.4)	1.9 (1.6, 2.3)	1.1 (0.8, 1.4)	<0.001	<0.001	<0.001

Abbreviations: NRS, Numeric Rating Scale; SPPB, Short Physical Performance Battery; TUG, Timed Up & Go.

Between 3 and 12 months, there were further statistically significant improvements in SPPB total score (*p* < 0.001) (increased by 0.7 points), TUG (*p* < 0.001) (decreased by 1.7 s), stair climbing test (*p* < 0.001) (decreased by 5 seconds), EQ‐5D‐5L Index (*p* < 0.001) (decreased by 0.2), and pain in activity (*p* < 0.001) (decreased by 0.8 points). There were no statistically significant improvements in EQ‐5D‐5L VAS score or pain at rest during this interval (*p* > 0.05) (Table 2; Figure 2).

#### 3.1.2 Recovery of physical function, HRQoL, and pain for those discharged to nursing home placement

There were statistically significant improvements in SPPB total score (*p* < 0.001) (increased by 3.6 points), TUG (*p* < 0.001) (decreased by 41.5 s), EQ‐5D‐5L index (*p* < 0.001) (increased by 0.1 index), EQ‐5D‐5L VAS score (*p* < 0.001) (increased score by 9 mm), and NRS Pain in activity (*p* < 0.001) (decreased by 3.3) (*p* < 0.001) between discharge to 3 month assessment. There were no statistically significant improvements in NRS Pain at rest during this interval (*p* > 0.05) (Table 3; Figure 2).

**TABLE 3:** Changes in physical health outcomes after hip fracture and change differences between the groups reported per protocol

	Participants transferred to a short‐term nursing home placement after hospital stay (*N* = 140)			Participants transferred directly home after hospital stay (*N* = 67)			Differences in change (*p*‐value) from T1 to T2	Differences in change (*p*‐value) from T2 to T3
	Mean (95% CI)			Mean (95% CI)				
	T1	T2	T3	T1	T2	T3		
SPPB total 0–12	2.9 (2.5, 3.3) (*n* = 140)	6.5 (5.8, 7.2) (*n* = 125)	7.2 (6.5, 7.9) (*n* = 99)	4.6 (4.0, 5.1) (*n* = 67)	9.3 (8.5, 10.0) (*n* = 55)	9.8 (9.1, 10.6) (*n* = 53)	<0.005^a^	0.85
SPPB balance 0–4	1.8 (1.5, 2.1) (*n* = 140)	2.8 (2.5, 3.0) (*n* = 125)	2.7 (2.5, 3.0) (*n* = 99)	2.9 (2.5, 3.3) (*n* = 67)	3.4 (3.1, 3.7) (*n* = 55)	3.4 (3.1, 3.7) (*n* = 53)	0.03^b^	0.62
SPPB walking speed 0–4	1.1 (0.9, 1.2) (*n* = 140)	2.7 (2.5, 2.9) (*n* = 125)	2.9 (2.7, 3.1) (*n* = 99)	1.5 (1.3, 1.7) (*n* = 67)	3.5 (3.2, 3.7) (*n* = 55)	3.6 (3.3, 3.8) (*n* = 53)	0.09	0.47
SPPB strength 0–4	0.02 (−0.05, 0.1) (*n* = 140)	1.0 (0.7, 1.3) (*n* = 125)	1.5 (1.2, 1.9) (*n* = 99)	0.2 (0.1, 0.3) (*n* = 67)	2.3 (1.9, 2.7) (*n* = 55)	2.7 (2.3, 3.1) (*n* = 53)	<0.001^a^	0.93
TUG, seconds	57.9 (51.2, 64.5) (*n* = 68)	16.4 (14.5, 18.3) (*n* = 114)	14.3 (12.6, 16.0) (*n* = 98)	38.0 (31.2, 44.8) (*n* = 54)	10.9 (8.9, 12.9) (*n* = 55)	9.6 (7.9, 11.3) (*n* = 53)	<0.005^b^	0.76
Stair climbing test, seconds	‐	26.4 (22.0, 30.9) (*n* = 86)	20.3 (17.0, 23.6) (*n* = 76)	‐	15.9 (12.5, 19.4) (*n* = 53)	12.5 (11.0, 14.1) (*n* = 52)	‐	0.20
EQ‐5D‐5L								
Index	0.4 (0.4, 0.5) (*n* = 137)	0.5 (0.5, 0.6) (*n* = 119)	0.7 (0.6, 0.8) (*n* = 95)	0.5 (0.4, 0.6) (*n* = 62)	0.5 (0.4, 0.6) (*n* = 40)	0.8 (0.7, 0.8) (*n* = 53)	0.10	0.14
Health score 0–100	50.5 (46.5, 54.6) (*n* = 134)	59.5 (56.1, 62.9) (*n* = 120)	61.8 (57.7, 65.9) (*n* = 102)	56.5 (51.1, 61.9) (*n* = 62)	72.3 (67.7, 76.7) (*n* = 56)	74.7 (69.2, 80.1) (*n* = 54)	<0.001^a^	<0.001^a^
Numeric rating scale								
Pain in rest	1.0 (0.6, 1.3) (*n* = 138)	1.0 (0.7, 1.4) (*n* = 120)	0.7 (0.4, 1.0) (*n* = 102)	1.6 (1.1, 2.1) (*n* = 62)	0.9 (0.5, 1.4) (*n* = 56)	0.7 (0.3, 1.1) (*n* = 54)	0.09	0.71
Pain in activity	5.2 (4.7, 5.6) (*n* = 134)	1.9 (1.5, 2.3) (*n* = 120)	0.9 (0.5, 1.3) (*n* = 101)	4.8 (4.2, 5.4) (*n* = 62)	2.0 (1.4, 2.5) (*n* = 56)	1.5 (1.0, 1.9) (*n* = 54)	0.12	0.15

*Note*: T1 = baseline, *T*2 = 3 months after hip fracture, *T*3 = 12 months after hip fracture.

Abbreviations: SPPB, Short Physical Performance Battery; TUG, Timed Up & Go.

^a^ Statistically significant in favour of patients transferred directly to home after hip fracture.

^b^ Statistically significant in favour of participants transferred to a short‐term nursing home placement after hip fracture.

There were further statistically significant improvements in SPPB total score (*p* < 0.001) (increased by 0.7 points), stair climbing test (*p* < 0.001) (decreased by 6.1 s), EQ‐5D‐5L index (*p* < 0.001) (increased by 0.2 index), and NRS Pain in activity (*p* < 0.001) (decreased by 1.0 point) between 3‐to 12 month follow‐up (Table 3; Figure 2).

#### 3.1.3 Recovery of physical function, HRQoL, and pain for people discharged directly home following hip fracture

There were statistically significant improvements in SPPB total score (*p* < 0.001) (increased by 4.7 points), TUG (*p* < 0.001) (decreased by 27.1 s), EQ‐5D‐5L health score (*p* < 0.001) (increased by 15.8 mm), and NRS Pain at rest and in activity (*p* < 0.001) (decreased by 0.7 points at rest and 2.8 points in activity) from discharge to 3 months follow‐up. There were further statistically significant changes in SPPB total score (*p* < 0.001) (increased by 0.5 points) and stair climbing test (*p* < 0.001) (decreased by 3.3 s between the 3 and 12 month follow‐up intervals; Table 3; Figure 2).

#### 3.1.4 Differences in scores during 12 months between those discharged directly home versus home via nursing home placement

A summary of the differences in outcome scores at each time‐point are presented in Table 3. Those discharged directly home after hip fracture reported greater change in SPPB total score between discharge to 3 months (1.1. points; *p* < 0.005) compared to participants discharged to a short‐term nursing home placement. There was a substantial change in EQ‐5D‐5L VAS score between discharge and 3 months for those discharged directly home compared to nursing home placement (6.8 points; *p* < 0.001). Conversely, participants discharged to a short‐term nursing home demonstrated greater change in TUG score from discharge to 3 months (14.4 s; *p* < 0.005) (Table 3). There were no statistically significant differences in change between groups in the reported outcome measures from 3 to 12 months, except in EQ‐5D‐5L health score (*p* < 0.001) in favour of those who transferred directly home post‐discharge (Table 3).

### 3.2 Predictors of SPPB scores at 3 and 12 months after hip fracture

The baseline characteristics and clinical outcomes, which correlated statistically significant with SPPB at 12 months after hip fracture are presented in Supporting Information S1. Male sex, the need of a walking aid outdoors prior to the fracture, handgrip strength, and SPPB total score at baseline were identified as predictors of SPPB total score at 3 months (*p* < 0.05). Age, the need of a walking aid both indoors and outdoors prior to the fracture, and SPPB total score at baseline were identified as predictors of SPPB total score at 12 months after hip fracture (*p* < 0.05) (Table 4). The total model explained 52% of the variance in the SPPB total at 3 months (adjusted *R* ^*2*^ = 0.52) and 59% of the variance in the SPPB total at 12 months after surgery (adjusted *R* ^*2*^ = 0.59).

**TABLE 4:** Acute phase predictors of physical function (SPPB) at 3 (*n* = 173) and 12 (*n* = 118) months in participants after hip fracture

Variables	Crude estimates			Adjusted estimates		
	*β*	95% CI	*p*	*β*	95% CI	*p*
SPPB total at 3 months						
Age	0.002	−0.07, 0.08	0.95	‐	‐	‐
If male	−1.3	−2.8, 0.2	0.09	−1.5	−2.6, −0.38	0.009
BMI	−0.02	−0.06, 0.02	0.30	‐	‐	‐
Higher education	0.05	−0.41, 0.29	0.73	‐	‐	‐
In need of a walking aid indoors prior fracture	0.39	−1.3, 2.1	0.66	‐	‐	‐
In need of a walking aid outdoors prior fracture	1.5	0.002, 3.0	0.05	1.4	0.51, 2.4	0.003
In need of help from community nurses	−0.22	−0.72, 0.28	0.39	‐	‐	‐
Grip strength	0.05	0.02, 0.13	0.20	0.06	0.006, 0.11	0.03
NRS pain at rest	0.18	−0.11, 0.48	0.22	‐	‐	‐
NRS pain during activity	−0.11	−0.36, 0.14	0.37	‐	‐	‐
EQ index	0.07	−2.8, 3.0	0.95	‐	‐	‐
EQ health score	0.003	−0.02, 02	0.84	‐	‐	‐
TUG	−0.006	−0.02, 0.01	0.59	‐	‐	‐
SPPB total	0.71	0.36, 1.0	<0.001	0.78	0.53, 1.0	<0.001
SPPB total at 12 months						
Age	−0.05	−0.12, 0.007	0.08	−0.05	−0.11, −0.001	0.04
If male	−0.36	−1.8, 1.0	0.61	‐	‐	‐
BMI	−0.009	−0.04, 0.02	0.57	‐	‐	‐
Higher education	0.17	−0.47, 0.13	0.26	‐	‐	‐
In need of a walking aid indoors prior fracture	2.7	1.0, 4.3	0.001	1.7	0.54, 2.8	0.004
In need of a walking aid outdoors prior fracture	2.0	0.85, 3.2	0.001	1.6	0.67, 2.7	0.001
In need of help from community nurses	−0.18	−0.59, 0.21	0.36	‐	‐	‐
Grip strength	0.007	−0.06, 0.08	0.83	‐	‐	‐
NRS pain at rest	0.03	−0.21, 0.27	0.78	‐	‐	‐
NRS pain during activity	−0.01	−0.24, 0.21	0.92	‐	‐	‐
EQ index	0.05	−2.3, 2.4	0.96	‐	‐	‐
EQ health score	−0.001	−0.02, 0.02	0.93	‐	‐	‐
TUG	−0.01	−0.02, 0.009	0.30	‐	‐	‐
SPPB Total	0.37	0.07, 0.66	0.01	0.6	0.39, 0.87	<0.001

*Note*: Unstandardised beta, 95% CI, and *p* value given for crude and adjusted estimates in a multiple regression analysis. The total model explained 52% of the variance in the SPPB total at 3 months (adjusted *R* ^*2*^ = 0.52) and 59% of the variance in the SPPB total at 12 months after surgery (adjusted *R* ^*2*^ = 0.59).

Abbreviations: BMI, Body mass Index; NRS, Numeric Rating Scale; SPPB, Short Physical Performance Battery; TUG, Timed Up & Go.

## 4 DISCUSSION

This study reports the recovery of physical function, HRQoL, and pain of participants after hip fracture. The results indicate a gradual improvement in physical function, HRQoL and pain during the initial 12 months post‐fracture. The largest improvements in physical function occur within the first 3 months for both those in need of a short‐term nursing home placement and those transferred directly home. The strongest predictors of physical function at 3 and 12 months post‐fracture were physical function at hospital discharge and pre‐fracture requirement of a walking aid.

Previous literature has reported that health status at the time of hip fracture has an impact on the patients' recovery of physical function (Beaupre et al., 2013). This is supported by our findings for both the total cohort and when reported by hospital discharge destination. As may be expected, our findings suggest that those who require a short‐term nursing home placement after hip fracture have poorer physical function than those discharged directly home. Individuals who were discharged directly home reported better improvements in SPPB total score and EQ health score after 3 months. However, this subgroup had a higher start‐point in physical function, and therefore the reported higher endpoint scores in physical function after three 3and 12 months may be expected.

The largest improvements in physical function occurred within the first 3 months after hip fracture. This contrasts to previous findings where improvements in physical function occurred up to 6‐ (Fukui et al., 2012) or 12 months (Magaziner et al., 2000; Vochteloo et al., 2013) post‐hip fracture. The characteristics between these cohorts and our study are similar, and it is difficult to find credible explanations to the differences between the results in these studies.

One question is whether the improvements in physical function after 3 and 12 months for the total sample and between the groups are clinically meaningful changes. According to Perera et al. (2006) a meaningful clinical change in SPPB is 0.5 point. Our results show that there were substantial improvements with clinical relevance after 3 and 12 months for both groups as well as for the total sample.

The results indicate that physical function at hospital discharge can predict physical function at 3 and 12 months post‐hip fracture. These findings are supported in previous cohorts of older people discharged from an acute care hospital settings (Corsonello et al., 2012; Volpato et al., 2011). Furthermore, low SPPB scores have been shown to predict poor outcomes, such as new falls, mobility loss, disability, hospitalisation, a longer hospital stay, nursing home admission, and death (Bean et al., 2002; Guralnik et al., 1994, 1995, 2000; Pavasini et al., 2016; Penninx et al., 2000; Volpato et al., 2011). In addition, previous research suggests that the SPPB can detect the early stages of frailty (Verghese & Xue, 2010), and that a total score ≤9 points can distinguish frail from non‐frail individuals (da Câmara et al., 2013). Therefore, the findings in this study can be useful in helping clinicians to identify those at risk of a poor outcome and set attainable therapeutic goals.

Reduced handgrip strength has previously been shown to be the strongest predictor of poor physical function after hip fracture (Xu et al., 2019). In this study, handgrip strength was a predictor of physical function at 3, but not at 12 months. Low handgrip strength from as early as midlife and onwards may be related to reduced future physical disability (den Ouden et al., 2011; Lee et al., 2018) and premature mortality (Celis‐Morales et al., 2018; Strand et al., 2016). This provides further evidence that it can be a useful marker of healthy ageing (Sayer & Kirkwood, 2015), as well as a feasible prognostic tool in clinical assessment (Lara et al., 2015).

We report that the use of walking aids pre‐fracture was a strong predictor of physical function at both 3 and 12 months post‐hip fracture. The patients' need of a walking aid pre‐fracture is infrequently evaluated in hip fracture research (Heikkinen & Jalovaara, 2005). This new and important finding is of great clinical value. The result may suggest that healthcare professionals may use the patients' need of a walking aid pre‐fracture to identify those at risk of poor long‐term functional outcomes rather than expecting them to undergo more physically demanding assessments, such as the SPPB immediately prior to discharge. Furthermore, clinicians can use this knowledge to identify patients at high risk of poor outcome after hip fracture. It may also be used to identify people pre‐fracture to provide rationale to intervene through rehabilitation strategies and falls‐prevention interventions to mitigate poorer outcome if a hip fracture were to occur.

### 4.1 Study limitations

Whilst this study provides new and novel insights to aid hip fracture rehabilitation, several important limitations should be considered. First, we only measured outcomes at 3 time points. It therefore remains uncertain whether the changes occurred early or later within this 9 month intervals or whether they occurred gradually. Whilst we could have assessed the outcomes at 3 months intervals during the first postoperative year, this was not performed to avoid overburden participants, whilst providing data on time points where change would be expected.

Secondly, a measurement floor‐effect may have occurred due to participants' physical impairments, especially in the first assessment point. This has been previously reported in mobility tests, such as TUG typically in an older acute medical population (de Morton et al., 2007). Accordingly, the ability to discriminate outcome between participants at this time point may have been reduced. Finally, the sample were drawn from one hospital and two associated municipalities in Norway. Whilst we believe this provides a representative sample for this country, generalising to other developed and developing cohorts and different health service provision, may be questioned.

## 5 IMPLICATIONS FOR PHYSIOTHERAPY PRACTICE

The largest improvements in physical function, HRQoL, and pain occur within the first 3 months following hip fracture. Those in need of a short‐term nursing home placement have worse physical function than individuals discharged home from hospital. The use of a walking aid prior to hip fracture and physical function prior to hospital discharge are strong predictors of physical function at 3 and 12 months following hip fracture. Health professionals may use the patients' need of a walking aid pre‐fracture to identify patients at risk of poor long‐term functional outcomes rather than to use more physically demanding assessments, such as the TUG in the early phase after hip fracture. This may contribute to avoidance of floor‐effect in physically demanding assessments for the most fragile patients. Asking patients whether they used a walking aid prior to hip fracture may therefore be valuable to provide an indicator on rehabilitation requirements and expected recovery.

## CONFLICT OF INTEREST

There are no conflicts of interests.

## ETHICS STATEMENT

This trial was approved by the Reginal Committee for Ethics in Medical Research (South‐East Norway) (2015/2147).

## Supporting information

Supporting Information S1

## Data Availability

The data that support the findings of this study are available from the corresponding author upon reasonable request.
