# Supplementary material for: Recovery and prediction of physical function 1 year following hip fracture
Source: Physiother Res Int. 2022 Mar 24;27(3):e1947. doi: 10.1002/pri.1947 (PMC9541337; doi:10.1002/pri.1947)
Supplement: Supplementary file 1 — Supporting Information S1 [file PRI-27-e1947-s001.docx]

Supplementary file 1. Bivariate correlation matrix between SPPB at 12 months after hip fracture and control variables at baseline

| **Variables** | SPPB 12 months | SPPB | TUG | EQ health score | EQ Index | Pain during activity | Pain at rest | Grip strength | Help from community nurses | Walking aid outdoors | Walking aid indoors | Higher education | Sex | BMI |
| --- | --- | --- | --- | --- | --- | --- | --- | --- | --- | --- | --- | --- | --- | --- |
| Age | -.53** | -.52** | .24** | -.12 | -.15* | .09 | -.11 | -.42** | .24** | -.52** | -.47** | -.02 | .005 | -.03 |
| BMI | -.002 | -.02 | -.02 | -.01 | -.05 | -.05 | -.05 | .08 | -.07 | .08 | -.04 | -.04 | .03 |  |
| If male | -.04 | -.03 | -.07 | .05 | .05 | -.06 | -.15* | .55** | .01 | -.008 | -.09 | .12 |  |  |
| Higher education | .16* | .08 | .000 | .12 | .11 | .05 | .01 | .15 | -.10 | .15* | .15* |  |  |  |
| Walking aid indoors | .61** | .47** | -.14 | .22** | .22** | -.20** | -.09 | .29** | -.39** | .70** |  |  |  |  |
| Walking aid outdoors | .66** | .57** | -.04 | .23** | .18** | .003 | -.12 | .32** | -.30** |  |  |  |  |  |
| Help from community nurses | -.30** | .22** | .09 | -.23** | -.15* | .15* | .06 | -.32** |  |  |  |  |  |  |
| Grip strength | .33** | .33** | -.27** | .15* | .22** | -.10 | -.16* |  |  |  |  |  |  |  |
| Pain at rest | .02 | -.008 | -.02 | -.06 | -.25** | .35** |  |  |  |  |  |  |  |  |
| Pain during activity | -.19* | .17* | .19* | -.16* | -.41** |  |  |  |  |  |  |  |  |  |
| EQ Index | .28** | .28** | -.17 | .34** |  |  |  |  |  |  |  |  |  |  |
| EQ health score | .17* | .18* | -.009 |  |  |  |  |  |  |  |  |  |  |  |
| TUG | -.31** | -.48** |  |  |  |  |  |  |  |  |  |  |  |  |
| SPPB | .66** |  |  |  |  |  |  |  |  |  |  |  |  |  |

BMI= Body Mass Index, TUG= Timed Up & Go, SPPB= Short Physical Performance Battery. *Pearson’s correlation coefficient; *P* <.01, **Pearson’s correlation coefficient; *P* <.05
